# Hearing effects from intermittent and continuous noise exposure in a study of Korean factory workers and firefighters

**DOI:** 10.1186/1471-2458-12-87

**Published:** 2012-01-27

**Authors:** In-Sung Chung, Isabella M Chu, Mark R Cullen

**Affiliations:** 1 Division of Occupational and Environmental medicine, Department of Preventive medicine, Keimyung University School of Medicine, 216 Dalseong-ro, Jung-gu, Daegu, South Korea, 700-712; 2 Stanford University School of Medicine, General Medical Disciplines, 1265 Welch Road, MSOB X-338, Stanford, CA 94305-5411, USA

## Abstract

**Background:**

South Korea and surrounding countries in East Asia are believed to have the highest proportion in the world of high frequency hearing loss due to occupational noise exposure, yet there has been limited information published in international journals, and limited information for control of noise in local workplaces beyond strategies from western countries. We exploit medical surveillance information from two worker groups to enhance local knowledge about noise-induced hearing loss and explore the possible importance of shift work to risk.

**Methods:**

Four-years of hearing data were evaluated for 81 male farm machine factory workers and 371 male firefighters who had successfully completed a health examination and questionnaires for the duration of the study period. The averages of hearing thresholds at 2, 3, and 4 kHz were used as the primary end-point for comparison. Repeat measure analysis adjusted for age, exposure duration and smoking status was used to measure the difference in hearing threshold between the two groups.

**Results:**

Noise levels were measured in the factory at a mean of 82 dBA, with a range of 66-97. No concurrent measurements were taken for the firefighters, but historic comparison values showed a wider range but a similar mean of 76-79 dBA. Although losses during follow-up were negligible, the factory workers had significantly (*P *< 0.0001) more hearing loss at the baseline of the study than the firefighters in both ears at 2, 3, and 4 kHz, adjusted for age, duration of employment and smoking status. Among those with 10 years of employment, mean losses at these frequencies among the factory workers fell into the impairment range (> 25 dB loss). Firefighters also showed increased losses associated with longer exposure duration, but these were significantly less marked. Losses at lower frequencies (< or = 1 kHz) were negligible in both groups.

**Conclusions:**

Korean work environments with continuous noise exposure in the measured range should consider implementation of a hearing conservation program. Further evaluation of hearing loss in workers exposed to irregular or intermittent high noise levels, such as firefighters, is also warranted.

## Background

Rapid expansion and change in Asian economies has resulted in increasing numbers of workers exposed to high intensity noise. However, in many countries, regulation and control lag behind Japan, Australia and New Zealand and nations in Western Europe and North America. A recent analysis of adult hearing loss [1] concluded that China, Mongolia and South Korea have the highest proportion of sensorineural hearing loss attributable to occupational noise of any region in the world.

In Korea, work-related noise induced hearing loss (NIHL) is defined as hearing levels above 30 dB for the average 0.5, 1 and 2 kHz and 50 dB at 4 kHz [2]. Despite systemic approaches to prevent NIHL such as a NIHL surveillance system with periodic annual audiometric examinations for workers exposed to a mean equivalent sound levels of 85 dBA time weighted average (TWA) based on 40 h per week [2] and a hearing-conservation program published by the Ministry of Employment and Labor [3] Korean workers in noisy industries remain at high risk of acquiring NIHL [4,5]. As of 2009, NIHL is the leading occupational disease [6], constituting 94.8% of all work-related diseases in South Korea [4]. The primary reason for this is that almost all regulations only establish actions to be taken regarding noise monitoring and hearing protection. There are virtually no regulations regarding training (employer and employee), control of the source of noise and other factors which may affect progression to NIHL such as work schedule.

Despite the magnitude of the problem little research has been published in the international literature regarding how best to control this problem in the local environment [5]. One aspect that may merit investigation in the East Asian context is the impact of work schedule on hearing loss, particularly the duration and frequency of non-exposure periods between shifts. Work schedules may differ by the time of day (i.e. day, evening, night), fixed versus rotating schedules, direction of rotation, number of hours worked per week, number of consecutive days worked, number of rest days, and so on. Chou et al. reported recently that working 12 h a day for two consecutive days and then having two days off resulted in a lower degree of hearing loss than working an 8-h swing shift in a cross sectional study [7]. Additionally, the patterns of noise exposure during duty vary. Animal studies show that brief intervals of high noise exposure produces less temporary and permanent hearing loss and less cochlear damage than continuous exposures of equal energy and total duration [8,9].

As part of government mandated surveillance in South Korea, one of the authors (IS Chung) had the opportunity to collect and study hearing surveillance data collected in various work environments over the course of 4 years. Although the surveillance was designed for control purposes and not research per se, we present our findings to contribute further knowledge about noise induced hearing loss (NIHL) in this region and to provide some new empirical data on the effects on hearing loss by noise exposure pattern.

## Methods

### Study population

Surveillance, including audiograms and a questionnaire, was conducted in a farm machine factory as a continuous regular noise exposure group and among firefighters as an example of an intermittent, irregular noise exposure group for 4 years. Data from 81 male farm machine factory workers and 371 male firefighters who completed a routine annual health exam for 4 years and had no history of ear-related illness were chosen for the final analysis. Although change during the study period was not the focus of the study, data from subjects who had not completed four consecutive annual health exams were excluded to assure a stable group of workers for the study and to compensate for any year to year variation in testing conditions (see below). The medical history questionnaire, "Checkup List for Regular Health Examination," (Additional File 1) included an abbreviated medical history including current symptoms, past and present medical conditions and health risk behaviors such as exercise, eating, drinking and smoking habits. The "Noise-induced Hearing Loss Work-up Sheet" (Additional File 2) included questions about the history of noise exposure at their past and current job and about non-occupational sources of noise including noisy hobbies or other noisy jobs and other medical factors that could contribute to hearing loss at the time of a hearing test. Farm machine factory workers worked 8-h for 5 weekdays and firefighters worked for 9 h for two consecutive days and a 15 h night shift for two consecutive days, followed by 2 days off.

Permission to conduct this study was granted by the Stanford University Administrative Panel on Human Subjects in Medical Research.

### Noise monitoring and hearing examination

Noise exposure levels in the farm machine factory were evaluated by work site environmental monitoring using CR 110A dosebadge (Cirrus, UK) attached to individuals. The number of samples depended on the total number of workers at the time data were collected, with a minimum of one sample for every 5 workers each year. Measurements were taken on those who worked closest to noise sources to provide a "worst case" profile. Data were gathered from 31, 31, 32 and 28 workers at the same department of the factory during the 4 years of the study period. No comparable samples were obtained from the firefighters; instead we relied on historic published data on exposure.

Pure-tone air conduction hearing thresholds were measured at 0.5, 1, 2, 3, 4 and 6 kHz in both ears every year as a part of an annual routine health examination. The hearing test was administered by an occupational health nurse who had completed a certification course to administer the test. The hearing tests were performed in a sound proof audiometric test booth. The Occupational Health and Safety Agency recommendation to allow at least 14 h of noise-free time prior to hearing test administration was followed. The minimum time of presumed non-exposure was approximately 15 h. For the farm machinery factory workers hearing tests were conducted in the morning prior to their shift. Firefighters generally also took the test in the morning prior to initiating their shift, however in some cases, the test was administered on their day off. The annual health examination also included otoscopy for all workers. Data from workers with abnormal otoscopic findings were excluded from the analysis.

### Statistical analysis

Data were analyzed using SPSS version 16. A two-sample *t*-test was used to test differences between the study groups for quantitative parameters with hearing loss. The *χ*^2 ^test was used to examine differences between the study groups for demographic data. We measured the difference in hearing thresholds in the two groups using GLM repeat measure adjusted for smoking status (Yes/No), age (continuous) and duration of employment from the original date of hire. We separately analyzed hearing at 1 kHz and the average of hearing thresholds at 2, 3, and 4 kHz. The number of subjects reporting non-occupational exposure to high levels of noise (such as noisy hobbies) was negligible and this covariate was not included in our final analysis.

## Results

From the two worker groups we enrolled a total of 1225 workers during a 4-year period (2006-2009). The 452 workers with a completed audiometric examination for four consecutive years were included in the analysis (36.9%). The general characteristics of workers in each group are shown in Table 1. Based on data from 2006, the farm machine factory workers were significantly older than the firefighters; age 44.98 vs 39.46 years (*p *< 0.0001), more likely to smoke (*p *< 0.0001) and had a longer duration of employment in their present workplace (*p *< 0.0001).

**Table 1:** Demographic and behavioral characteristics of study subjects from the annual routine health exam and study questionnaire

		Firefighters	Farm Machinery Factory Workers	*P*-Values
Total (N)		371	81	--

Noise level (dBA)	Mean Range	76-79*	82.45	--
			
		68-115*	67-98	

Age at study initiation (2006)	Mean (S.D.)	39.46 (6.89)	44.98 (6.08)	< 0.0001

Alcohol (%)		222	50	< 0.0001
			
no		40.2	38.3	
			
yes		59.8	61.7	

Smoking (%)		66	34	< 0.0001
			
no		82.2	58	
			
yes		17.8	42	

Work period in months (2006)	Mean (SD)	134.01 (90.34)	256.20 (72.74)	< 0.0001

Duration of current employment	0-10 years	172 (46.36%)	3 (3.7%)	--
		
	11-20 years	155 (41.78%)	35 (43.2%)	
		
	> 20 years	44 (11.86%)	43 (53.1%)	

Shift length		9 or 15	8 or 10	--

* Historically reported values in Korea. See discussion for fuller description

The distribution of noise samples is shown in Table 2. The mean noise level for the farm machine factory via environmental monitoring was approximately 82 dBA with a range of 66-98 dBA and little year to year change. Notably, almost all the factory workers were issued, and observed to be using, hearing protection.

**Table 2:** Distribution of measured noise exposure in the farm machinery factory workers over the four year study observation period

	2006	2007	2008	2009
Number of samples	31	31	32	28

Mean dBA (SD)	82.48 (5.13)	82.44 (6.68)	82.46 (6.56)	82.41 (6.85)

Median dBA	82.7	83.2	83	82.3

Range dBA	70-96	66.8-92.6	66.8-97.3	71.6-98

Because of local regulations routine noise level sampling was not performed among the firefighters. Historic data from Korea suggests mean exposures in the range of 76-79 dBA [10]. Previous studies in the U.S suggest that the range of exposures is large, with reports of up to 110-115 dBA during actual fire emergencies [11]. Hearing protection was not generally used in this group.

The two groups differed significantly in their total mean duration of work. The firefighter's mean total work period was 134.01 months compared to the farm machine factory's workers mean of 256.20 months (*p *< 0.05). Table 1 describes the difference in distribution of employment by 10-year strata.

**Figure 1:**
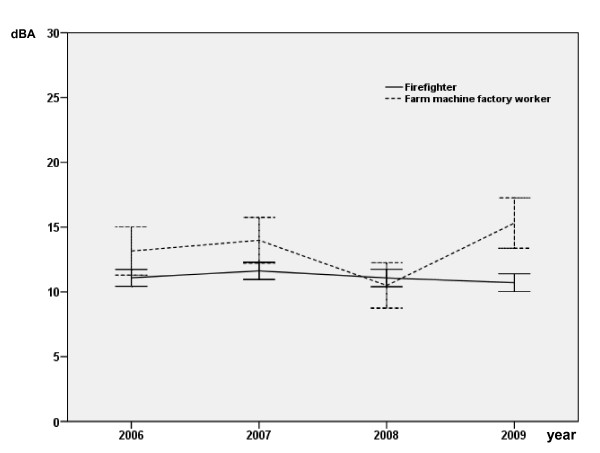
**Mean hearing level (dB) of left and right ear combined at 1 kHz for the four study periods**.

**Figure 2:**
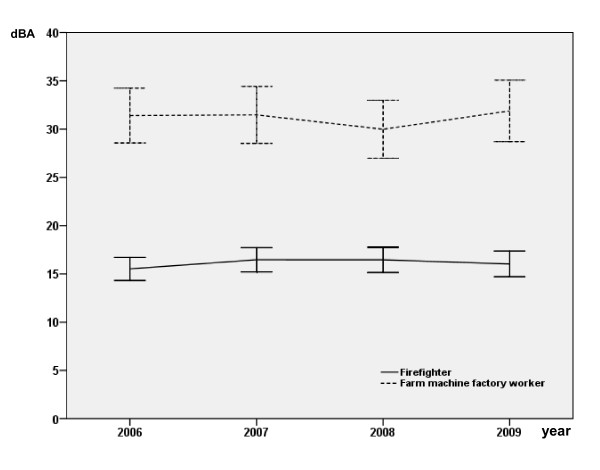
**Mean hearing level (dB) of left and right ear combined for 2, 3 and 4 kHz (combined) for the four study periods**.

Follow-up on hearing levels for 4 consecutive years is shown in Table 3 and Figures 1 and 2. There is no difference between the two groups at 1 kHz over the 4 years (Figure 1). Farm machine factory workers showed a significantly higher level of hearing loss than the firefighters when the mean hearing levels at 2, 3, and 4 kHz were compared (*p *< 0.0001; Table 3 and Figure 2). There was no significant year to year change in either group. Table 4 illustrates the difference in change of hearing level stratified by work duration. Differences are suggested even in the lowest duration group, but become more marked and statistically significant after 10 years employment (*p *< 0.0001). Age, duration of employment and cohort significantly impacted hearing in the 2-4 kHz frequency range in the simple regression, however in the multiple regression model, duration of employment and smoking were no longer significant (Tables 5 and 6).

**Table 3:** Follow up in hearing level (dB) at the average of 2, 3 and 4 kHz across the four years of the study period

		2006	2007	2008	2009
Left ear	Firefighter	15.93 ± 12.51	17.08 ± 13.43	16.95 ± 13.79	16.46 ± 14.09

	Factory worker	32.84 ± 14.41	33.21 ± 15.06	31.56 ± 15.98	33.60 ± 17.00

Right ear	Firefighter	15.10 ± 12.72	15.84 ± 13.25	15.95 ± 13.74	15.59 ± 14.15

	Factory worker	29.96 ± 13.48	29.73 ± 14.06	28.40 ± 13.66	30.16 ± 14.46

**Table 4:** Follow up of hearing level (dB) at the average of 2, 3 and 4 kHz across the four study periods stratified by work duration in years

Years at job			2006	2007	2008	2009	*P*-value
≤ 10							0.257

		Firefighter	13.11 ± 10.69	13.74 ± 11.10	13.53 ± 11.57	12.75 ± 11.92	

		Factory worker	24.72 ± 13.47	22.50 ± 15.07	20.83 ± 15.21	23.33 ± 15.43	

10 - ≤ 20							< 0.0001

		Firefighter	16.56 ± 11.67	17.31 ± 12.44	17.57 ± 13.01	17.28 ± 13.06	

		Factory worker	29.07 ± 13.44	28.10 ± 12.93	26.76 ± 13.72	28.57 ± 14.80	

> 20							0.001

		Firefighter	21.80 ± 13.22	24.72 ± 13.61	24.85 ± 13.44	25.27 ± 13.52	

		Factory worker	33.76 ± 12.06	34.85 ± 12.97	33.24 ± 12.76	35.17 ± 13.50	

**Table 5:** Coefficients and confidence intervals for all covariates shown by year using simple regression.

	2006		2007		2008		2009	
	**β**	**CI**	**β**	**CI**	**β**	**CI**	**β**	**CI**

Age	0.703	0.514-0.865	0.782	0.616-0.947	0.777	0.609-0.945	0.863	0.688-1.038

Work duration	0.055	0.043-0.066	0.059	0.047-0.071	0.058	0.046-0.070	0.065	0.053-0.077

Smoking status(no/yes)*	2.694	-0.293-5.68	1.493	-1.603-4.589	2.053	-1.071-5.178	1.445	-1.843-4.733

Group (Firefighter/Factory worker)**	15.887	13.028-18.747	15.012	11.992-18.032	13.528	10.407-16.649	15.858	12.644-19.073

Age, duration and group (firefighter or farm machinery factory worker) were all statistically significant

* Reference group is "No."

** Reference group is firefighters

**Table 6:** Coefficients and confidence intervals for all covariates using multiple regression for the first year of observations (2006).

	2006	
	**β**	**Confidence Interval**

Age	0.458	0.15-0.767

Work duration	0.004	-0.02-0.028

Smoking status (no/yes)*	0.983	-1.695-3.661

Group (Firefighter/Factory worker)**	12.544	9.196-15.892

Age and group (firefighter or farm machinery factory worker) remained significant in the final model

## Discussion

This study showed that day-shift work with continuous 8-h noise exposure in farm machinery factory workers produced higher level of hearing loss at 2, 3, and 4 kHz in both ears than nonstandard shift work in the comparison firefighters group. This finding was statistically significant after a 10-year work history in the stratified analysis, though duration was not significant in the multivariate model including age.

The most obvious explanation for the difference in risk is differential noise exposure. While we have documented exposures among farm factory workers in excess of 85 dBA, we do not have data to make a direct comparison with firefighters. However, a previous study which evaluated noise exposure among Korean firefighters according to their time-dependent activity patterns reported that firefighters were exposed to a mean noise level of 76 dBA during work time [10]. This result was similar to another study of 16 firefighters by Bryan et al. which reported a mean of noise exposure level of 78.7 dBA [12]. Based on the data available, the mean level of noise exposure for both groups evaluated may be reasonably comparable, but the pattern and duration of continuous noise exposure is likely very different.

The exposure pattern of the two groups differed in that farm machine factory workers were continuously exposed to a relatively constant level of noise during work time and the noise exposure of the firefighters usually varied by activity. Lee reported that firefighters spent 67% of total work time in "inside" areas at the fire station, including offices and waiting rooms and 23% of their time outside the station attending to fires and emergencies or transporting to and from these events. The noise exposure levels for inside and outside areas were 65-72 dBA and 79-85 dBA respectively [10]. The data we collected via questionnaire were consistent with these findings. Firefighters included in this study spent 20-30% of total work time in "outside" areas. Of this time, a portion was spent on fire trucks being transported to fires or other emergencies. These trips occurred approximately 10 times per duty day and lasted between 10-20 min per trip. The sources of noise exposure in the outside areas were fire engines, horns/sirens and pumps. Several noise surveys conducted by NIOSH to determine the magnitude of noise exposures among U.S. firefighters found that exposure levels varied from low to intense exposures according to OSHA or NIOSH noise criteria [13]. Firefighters traveling in emergency vehicles were exposed to noise ranging from 103.4 to 114.5 dBA. Mechanical equipment used by firefighters can produce up to 115 dBA with a mean duration of 30 min [14,15]. Though these are very high levels of exposure (over 90 dBA), the duration of this exposure was less than 10% of total work time for the firefighter group. Globally, firefighter's exposure to noise was intermittent in both intensity and duration, with both factors dependent on emergency codes during their shift. The range of noise exposure varied from 65-115 dBA. This intermittent exposure pattern is very different from the continuous noise at a relatively fixed level experienced by the farm machine factory workers. It may be that continuous noise exposure carries a greater risk of hearing loss than intermittent exposure even if the mean range in dBA is similar.

Shift schedules also differ between the groups. The shift length of farm machine factory workers was 8 h per day, 5 days per week, so non-exposure time was 16 h per day with up to 63 continuous hours of exposure-free time on weekends. Firefighters were free from noise exposure for 9 h during night-work duty, twice a week. Noise free time for the twice weekly day-shift was 15 h and up to 48 h of noise free time during weekends. Insufficient time between work shifts to allow workers to recover from temporary hearing deficits may affect hearing level as temporary threshold shifts generally last 24 h or more after cessation of excessive noise exposure for employees who work regularly [16]. Other previous studies [7] report that hearing loss is entirely preventable by administrative controls such as periodic shift rotation and limiting exposure to noise when levels exceed 85 dBA. We replicate these findings in that farm machine factory workers who were not given sufficient time to recover from temporary threshold shift experienced a higher level of permanent threshold shift (hearing loss) than firefighters exposed to similar levels of occupational noise. The duration of non-exposure periods related to shift type may be a contributing factor to this discrepancy, though to be sure, studies controlling for other known risk factors for hearing loss and measuring both noise magnitude and duration as well as hearing loss both groups in an identical fashion, would have to be carried out. Clark and Bohl evaluated hearing loss in firefighters compared with age-matched, non-occupationally exposed groups of individuals and reported that firefighters are not at risk for occupational noise-induced hearing loss, even though they work nonstandard shifts and are occasionally exposed to high levels of noise [17]. Our results differ slightly in that firefighters who had worked 20 years or more showed statistically significant hearing loss compared to other subgroups of firefighters when age and other risk factors were controlled for, particularly at 4 kHz (Data not shown). A possible explanation is that firefighters in Korea do not generally wear hearing protection despite frequent exposure to noise levels over 90 dBA. Over the long term this may inflict hearing loss though it would not be detected in shorter term studies or studies which did not consider long duration separately. Occupational exposure to high heat at fires may also impact noise-induced hearing loss [18]. A NIOSH investigation reported that health hazards exist for firefighters and recommended steps to the department to reduce noise exposure to help prevent further hearing loss [13]. In the case of farm machine factory workers, mean hearing levels were over 25 dB despite being exposed to a *mean *noise level below the current accepted threshold of 85 dBA according to environmental noise exposure monitoring. This finding is consistent with previous studies on chronic exposure to moderately high amounts of occupational noise. Rabinowitz et al. reported that the majority of 10 dB standard threshold shifts occurred in workers whose calculated mean ambient noise exposures were less than or equal to 85 dBA [19]. This may be partially explained by the greater number of individuals employed in environments with noise levels below 85 dBA as well as a decreased likelihood of hearing protection at noise levels not deemed to be dangerous [19]. Hearing loss in farm machine factory workers appears to increase with duration of exposure. Both groups experienced work related hearing loss with a duration of work longer than 20 years though the factory workers showed a greater degree of hearing loss than the firefighters at this time point.

There are several limitations to this study beyond the lack of concurrent exposure assessment of the firefighter group. Some factors which have been shown in the literature to affect noise-induced hearing loss such as alcohol consumption and the use of organic solvents were not controlled for. Although firefighters are exposed to mixed organic solvents during fire suppression [20], the exposure time is irregular and short, approximately 30 min per duty day, and fell below the NIOSH recommended occupational hazard threshold. Exposure to heat experienced during fire suppression may also be a significant risk factor for noise-induced hearing loss [18] and we did not control for this in our study. Only three farm machine factory workers reported a work period less than 10-years. Therefore, a much larger sample would be necessary to carry out a robust analysis of the effects of duration on hearing loss in employees with a work history of less than ten years. This merits investigation because there is evidence that there may be an initial, relatively rapid, phase of hearing loss, followed by a leveling off [21]. Previous research noted that 20% of firefighter audiograms showed threshold losses of 40-60 dB in hearing 3, 4, and 6 kHz test frequencies in one or both ears and 14% with still greater losses [17]. We used the average of hearing levels at 2, 3 and 4 kHz instead of high frequencies, which is the OSHA "recordable hearing loss" case definition and the standard metric of hearing loss progression.

Neither group showed evidence of decline in hearing during the 4 years of continuous observation. It is likely that the farm machinery factory workers were compliant in wearing hearing protection. The farm machinery factory hearing conservation program was formally monitored and the reported rates of adherence were approximately 85% over the study period. This is consistent with the literature which has noted a higher rate of hearing protection use in noisy industries [19]. The firefighters were noted to have very low rates of hearing protection use, so the absence of measured progression likely reflects either low exposure or the value of recovery from exposure between shifts.

A final important limitation which the above highlights is that the losses of interest for our study occurred in both populations largely before the observation began and we did not have sufficient data to control for previous occupational noise exposure. Though it is likely that both populations experienced considerable job stability due to the nature of the work environment in Korea and the size and stability of these employers, it does not follow that the level of noise exposure was consistent as assigned tasks may have changed during the course of employment. That having been said, it is certainly likely that hearing protection was used less regularly in the farm machinery workers before the hearing conservation program began, but the impact of this cannot be directly tested.

## Conclusions

As society ages, and life expectancies increase, the number of lifetime work years is also likely to increase. Consequently, the percentage of the population with long-term (20 years or more) exposure to occupational hazards, such as excessive noise, is likely to increase in tandem unless measures are taken to reduce risk.

Our data suggest that the current regulatory threshold of 85 dBA may not be sufficiently conservative. Typical work schedules are 5 days a week for 8 h a day which potentially do not allow adequate recovery time after each period of exposure. Work environments with levels of noise exposure close to 85 dBA should consider implementing a hearing conservation program. Further research is also needed on hearing loss for workers with long term duration of exposure as well as intermittent and irregular exposure to high levels of noise to determine optimal hearing conservation strategies for workers in these environments.

## Competing interests

The authors declare that they have no competing interests.

## Authors' contributions

ISC and MRC were involved in all aspects of the paper including conception and design of the study, acquisition, analysis and interpretation of data, drafting and revising the manuscript and approval of the final version. IMC made substantive intellectual contributions to the interpretation of the data, draft of the manuscript and final version for publication. All authors read and approved the final manuscript.

## Pre-publication history

The pre-publication history for this paper can be accessed here:

http://www.biomedcentral.com/1471-2458/12/87/prepub

## Supplementary Material

Additional file 1 **Checkup List for Regular Health Examination**. Click here for file

Additional file 2 **Noise-induced Hearing Loss Work-up Sheet**. Click here for file
