# Supplementary material for: Hearing effects from intermittent and continuous noise exposure in a study of Korean factory workers and firefighters
Source: BMC Public Health. 2012 Jan 27;12:87. doi: 10.1186/1471-2458-12-87 (PMC3280190; doi:10.1186/1471-2458-12-87)
Supplement: Additional file 1 — Checkup List for Regular Health Examination. [file 1471-2458-12-87-S1.PDF]

Checkup List for Regular Health Examination

Name of examinee		Resident registration number		**Telephone**	home:<br>mobile phone:
Name of workplace(No.)		Insurance card No.		E-mail address	
Address					zip code<br>-

※ **To ensure an accurate judgment of your health status, please do not leave any questions unanswered.**

1. Have you ever suffered from any of the following diseases or are you being treated for any of the following diseases or any other serious illness?

※ Tuberculosis, hepatitis, liver diseases, hypertension, stroke, diabetes, or cancer.

If you have any diseases, please list and describe below.

Name of diseases	Onset of diseases(year)	Current status	
		Complete recovery	Under treatment
(　　　)		①	②
(　　　)		①	②
(　　　)		①	②

2. Did any member of your family suffer or die from the following diseases?

① Liver diseases( )

② Hypertension ( )

③ Stroke ( )　④ Heart disease ( )

⑤ Diabetes ( )　⑥ Cancer ( )

3. Is there any kind of disease or symptoms which, you suspect, you might have?

① No　②Yes

(Name of disease :　　　　　　　　　　)

4. What kinds of food do you usually eat?

① I usually eat vegetables.

② I eat meat and vegetables equally.

③ I usually eat meat

5. How often do you drink alcohol?

① I never (rarely) drink.
② I drink twice or three times a month.
③ I drink once or twice a week.
④ I drink three or four times a week.
⑤ I drink almost every day.

6. When you drink, how much do you usually drink? (based on one bottle of Soju)

① less than a half bottle ② one bottle

③ one and a half bottles ④ more than two bottles

7. Do you smoke cigarettes currently?

① No, I don't

② I had smoked, but I quit smoking ___ year(s) ago.

③ Yes, I do

8. How many cigarettes do (did) you smoke per day?

① less than a half pack

② more than a half pack but less than one pack

③ more than one pack but less than two packs

④ two packs or more

9. How long have you been smoking?
If you quit smoking, how long did you smoke?

① less than 5 years ② 5~9years ③ 10~19years

④ 20~29years ⑤ more than 30years

⑥ the year when you started smoking :

10. How many times do you exercise to the point of perspiration (sweating) per week?

① never ② once or twice ③ three to four times ④ five to six times ⑤ everyday

11. Have you ever felt physical or mental difficulties over the past month?

① Frequently ② sometimes ③ never

④ I don't know
