# Supplementary material for: Hearing effects from intermittent and continuous noise exposure in a study of Korean factory workers and firefighters
Source: BMC Public Health. 2012 Jan 27;12:87. doi: 10.1186/1471-2458-12-87 (PMC3280190; doi:10.1186/1471-2458-12-87)
Supplement: Additional file 2 — Noise-induced Hearing Loss Work-up Sheet. [file 1471-2458-12-87-S2.PDF]

Noise-induced Hearing Loss Work-up Sheet

Occupational and Environmental medicine
Keimyung University School of Medicine

Date: - -

1. Last work time:____/_____AM/PM ___
2. Noise free time: total_____hours
3. Entering date: - -
4. Date of moving into current job: - -
5. Job history (Noise exposure): total ______years

Job title	Begin	End	Interval
(current)	- -	- -	months
	- -	- -	months
	- -	- -	months

6. Noise exposure history outside of work

   ☐ No ☐ Yes ( )

7. Protective equipment

   ☐ Ear plug ☐ Ear muff ☐ Not wearing

8. Frequency of use (for protective equipment)

   ☐ Never ☐ Intermittent ☐ Always

9. Army history:

Past personal history

10. Ear disease

    ☐ No ☐ Yes ( )

11. Other disease

    ☐ No ◻ Head trauma ◻ Syphilis ◻ Tuberculosis

    ☐ others ( )

Noise-induced Hearing Loss Work-up Sheet

Occupational and Environmental medicine
Keimyung University School of Medicine

Date: - -

Subjective symptoms

12. Is your social life disrupted because of difficulty hearing?

□ Not at all □ A little □ Same as other people □ Quite a bit □ Very much so

13. Do you turn the TV volume up?

□ Not at all □ A little □ Same as other people □ Quite a bit □ Very much so

14. Do you currently have any of the following symptoms?

☐ None ☐ Tinnitus ☐ Nervousness ☐ Indigestion ☐ Others(___________________)
